# Low-Salinity Rice-Straw Bioflocs Retain Nitrogen-Removal Capacity After Salinity Transfer and Improve Survival of Pacific White Shrimp

**DOI:** 10.3390/microorganisms14102349

**Published:** 2026-10-14

**Authors:** Dandan Huang, Qingqi Guo, Huilin Zhang, Shijun Chen, Zhaolin Lv, Zhaojie Deng, Canmin Yang, Lian Gan

**Affiliations:** 1 College of Marine Sciences, South China Agricultural University, Guangzhou 510642, China; huangdd@scau.edu.cn (D.H.); kuoqingqi@163.com (Q.G.); leng671494@163.com (H.Z.); csjscaucms@scau.edu.cn (S.C.); 15813347034@163.com (Z.L.); dengzhaojie@stu.scau.edu.cn (Z.D.); 13422163508@163.com (C.Y.); 2 Guangdong Province Engineering Research Centre of Aquatic Immunization and Aquaculture Health Techniques, South China Agricultural University, Guangzhou 510642, China; 3 Nansha– South China Agricultural University Fishery Research Institute, Guangzhou 511457, China

**Keywords:** *Penaeus vannamei*, *Vibrio harveyi*, ammonium chloride, nitrite, water quality, microbial community

## Abstract

Biofloc technology promotes dense microbial communities that remove nitrogenous waste and suppress pathogens, but the combined effects of salinity and carbon–nitrogen source selection on biofloc maturation and disease mitigation remain unclear. We evaluated two salinity levels (5 ppt and 25 ppt) and two carbon–nitrogen source combinations (rice straw with ammonium chloride or brown sugar with shrimp feed) in a 60-day biofloc trial. Under low salinity (5 ppt), the rice straw and ammonium chloride treatment reduced ammonia to undetectable levels by day 17 and nitrite to zero by day 40, while also establishing a stable, functionally diverse microbial community. When this mature biofloc was transferred abruptly to 15 ppt and 25 ppt, nitrite was again removed to zero within four days. Pre-inoculation of shrimp tanks with mature biofloc suppressed nitrite accumulation and lowered *Vibrio* loads in the hepatopancreas and gut, yielding a survival rate of 41% under natural infection compared with 0% in the control. In a *Vibrio harveyi* challenge, biofloc-treated groups achieved 90–93% survival versus 40–57% in non-inoculated groups and exhibited reduced tissue damage alongside increased abundance of beneficial *Bacillus*. Pre-stocking biofloc inoculation markedly reduces *Vibrio* infection risk and enhances shrimp survival, offering a cost-effective, eco-friendly strategy for improving water quality and biosecurity in high-density shrimp culture.

## 1. Introduction

*Penaeus vannamei* (Pacific white shrimp), valued for its rapid growth and favorable meat quality [1], ranks among the top three farmed shrimp species globally [2]. In 2024, global farmed production of this species reached approximately 7.7 million tons, the highest among all farmed aquatic animals [3]. China, as a leading producer, reported 2.1 million tons in 2022 [4]. Of this, 1.34 million tons originated from marine aquaculture, while 760,000 tons were produced in freshwater systems. Despite such production scales, the industry faces persistent challenges, including low unit yield and significant production variability, primarily due to disease outbreaks and water quality deterioration [5,6,7].

Water quality deterioration is a major concern in intensive aquaculture, primarily driven by the accumulation of total ammonia nitrogen (TAN) and nitrite (NO_2_^−^) [8]. Factors such as high stocking densities and organic waste buildup exacerbate nitrogen pollution, especially in systems with limited water exchange. Elevated TAN levels disrupt osmoregulation, damage gill tissues, and suppress immune-related gene expression in shrimp [9,10]. Similarly, nitrite exposure oxidizes hemoglobin to methemoglobin, impairing oxygen transport and causing hypoxia [11]. These physiological stresses increase shrimp susceptibility to bacterial infections, highlighting the critical need for effective water quality management [12,13].

*Vibrio* spp. are among the most virulent bacterial pathogens affecting Pacific white shrimp, causing significant disease outbreaks [14]. Infected shrimp typically exhibit tissue necrosis, muscle opacity, gut dysfunction, and high mortality rates [15,16,17]. Among these pathogens, *Vibrio parahaemolyticus* is the causative agent of acute hepatopancreatic necrosis disease (AHPND), which has spread rapidly from China since its initial outbreak in 2009, affecting major shrimp-producing countries and causing substantial economic losses [17]. Other pathogenic species, including *V. harveyi*, *V. alginolyticus*, and *V. cholerae*, also pose serious threats to shrimp health [18]. Water quality plays a pivotal role in modulating *Vibrio* pathogenicity. Elevated ammonia and nitrite levels compromise shrimp immunity and enhance *Vibrio* virulence, underscoring the importance of effective water quality management [19,20]. Recent studies have shown that nitrogen pollution upregulates virulence gene expression in *Vibrio* spp., thereby exacerbating disease severity. However, the mechanisms by which water quality deterioration influences *Vibrio–host* interactions remain poorly understood. Addressing this gap is essential for developing integrated strategies that simultaneously improve water quality and mitigate pathogen risks in high-density shrimp aquaculture.

Biofloc technology (BFT) has been increasingly adopted in intensive *P. vannamei* aquaculture as a sustainable strategy to improve water quality and production efficiency [21]. Its core principle involves adjusting the carbon-to-nitrogen (C/N) ratio to stimulate heterotrophic microorganisms, which assimilate inorganic nitrogen and reduce nitrogenous waste [7]. Maintaining a C/N ratio of 15 has been shown to accelerate biofloc formation, enhance nitrogen removal, and stabilize microbial community structure, thereby improving system resilience [22,23,24]. In addition to optimizing water quality, BFT influences microbial ecology, which plays a critical role in disease prevention. The biofloc matrix, which consists of heterotrophic bacteria, microalgae, zooplankton, and organic particulates, develops through exogenous carbon supplementation and promotes microbial metabolism and organic matter degradation [25]. By fostering beneficial microbial communities, BFT competitively inhibits pathogenic *Vibrio* spp. colonization and reduces the incidence of AHPND, ultimately improving shrimp health and survival rates [7,26].

Carbon and nitrogen sources are key factors shaping microbial metabolism, maintaining the carbon-to-nitrogen (C/N) balance, and stabilizing BFT systems [27]. Simple carbon sources, such as molasses and brown sugar, are readily metabolized by heterotrophic bacteria, promoting rapid microbial proliferation and nitrogen assimilation [19]. In contrast, complex carbon sources (e.g., straw, lignocellulose) require microbial degradation prior to utilization, resulting in slower biofloc formation but fostering more stable and resilient microbial communities [28]. Among various substrates, rice bran demonstrated superior efficacy in improving *P. vannamei* growth compared to molasses [29]. From both economic and environmental perspectives, utilizing low-cost agricultural byproducts such as straw, rice bran, wheat bran, and cassava can reduce production costs and enhance resource efficiency, particularly in regions like China where these materials are abundant [30]. Beyond carbon source selection, salinity is a critical environmental regulator of BFT performance. It affects not only shrimp growth but also biofloc formation and microbial community dynamics [31]. Under low-salinity conditions (≤5 ppt), microbial diversity tends to be higher, with heterotrophic bacteria dominating and facilitating efficient removal of TAN and NO_2_^−^ [32,33]. Conversely, high-salinity environments (15–30 ppt) promote the dominance of autotrophic bacteria prevalence, making nitrogen transformation more salinity-dependent and leading to slower yet more stable biofloc development [27,34]. While these individual effects are well documented, the interplay between carbon–nitrogen source selection and salinity, as well as its impact on biofloc maturation, nitrogen cycling, and microbial community succession, remains poorly understood. Bridging this knowledge gap is crucial for optimizing BFT applications across diverse aquaculture environments.

While BFT has shown promise in improving water quality and disease resistance, how salinity and carbon–nitrogen source selection jointly influence biofloc dynamics is still not fully understood. This study aims to investigate how varying salinity levels and carbon–nitrogen source strategies influence biofloc maturation, nitrogen cycling, and *Vibrio* suppression. In particular, we examine the potential of utilizing cost-effective agricultural byproducts as carbon sources to improve system sustainability. To achieve these objectives, we evaluated the effects of two salinity conditions (5 and 25 ppt) and two carbon–nitrogen source regimens (rice straw with ammonium chloride and brown sugar with shrimp feed) on nitrogen removal efficiency and the microbial community structure in BFT systems. We further assessed whether mature bioflocs cultivated under optimal conditions could maintain nitrogen removal performance following abrupt salinity changes. Additionally, we examined their preventive and curative effects against *Vibrio* infections in *P. vannamei*. By integrating water quality monitoring, microbial community analyses, and pathogen challenge tests, this work seeks to provide practical insights for developing economically viable BFT strategies that enhance both environmental sustainability and disease control in high-density shrimp farming.

## 2. Materials and Methods

### 2.1. Experimental Animals and Ethics Statement

Pacific white shrimp (*P. vannamei*) were purchased from Hainan Zhengtai Aquatic Breeding Co., Ltd. (Jiangmen, China). All experimental procedures were approved by the Animal Ethics Committee of the College of Marine Sciences, South China Agricultural University (approval number: 2021g029; approval date: 9 February 2021).

### 2.2. Biofloc Formation Under Varying Salinity Conditions

To investigate the effects of salinity on biofloc formation, bioflocs were cultured at two salinity levels: 5 parts per thousand (ppt) and 25 ppt. Two carbon–nitrogen source combinations were used: rice straw with ammonium chloride (STR group) and brown sugar with Pacific white shrimp feed (SUG group).

In the STR group, rice straw powder (20 mesh) was used as the carbon source, and ammonium chloride was used as the nitrogen source. Two salinity treatments were applied: STR_L (5 ppt) and STR_H (25 ppt). At the start of the experiment, *Lactobacillus plantarum* was added to the water at a concentration of 10^5^ CFU/mL. Ammonium chloride was not applied as a single pre-calculated dose. A C/N ratio of 15 was set as the formulation target for the system, and ammonium chloride was added intermittently, in small amounts, whenever the measured total ammonia nitrogen concentration indicated that additional nitrogen was required. The cumulative amount of ammonium chloride added over the cultivation period cannot be reconstructed from the available records, because the dosing log is incomplete and neither the initial charge nor the subsequent additions for the individual STR tanks can be fully verified.

In the SUG group, brown sugar (Chaozhou Chao’an District Lvyaf Food Factory, Chaozhou, China) was used as the carbon source, and Pacific white shrimp feed (No. 0, Guangdong Yuehai Feed Group Co., Ltd., Zhanjiang, China) served as the nitrogen source. Two salinity treatments were applied: SUG_L (5 ppt) and SUG_H (25 ppt). *Pediococcus* was added to the water at a concentration of 10^5^ CFU/mL at the start of the experiment and supplemented every three days for a total of four additions.

Both STR and SUG groups were replicated in triplicate under their respective salinity treatments, with a carbon-to-nitrogen (C/N) ratio of 15. The experiment was conducted in 12 cylindrical tanks (50 L, diameter 40 cm × height 37 cm), with each tank containing 40 L of water. The experiment lasted 60 days, during which continuous aeration was provided 24 h per day. Sodium bicarbonate (NaHCO_3_) was added as needed to regulate pH and total alkalinity, maintaining total alkalinity between 90–120 mg/L and pH within the range of 7.0–8.5.

Water samples were collected daily at 9:00 a.m. to measure ammonia nitrogen and nitrite nitrogen levels in the aquaculture system. Additionally, water samples were collected for 16S rDNA amplicon sequencing: STR_L group on days 20 and 40, STR_H group on days 20, 40, and 57, and SUG_L and SUG_H groups on days 40 and 53 when nitrite nitrogen levels had decreased to 0 mg/L.

Nitrate nitrogen was measured intermittently at selected cultivation days, rather than at every TAN and nitrite sampling time. Cultivation days were counted separately from the start of each STR and SUG experiment, with day 1 denoting the start of cultivation. Reportable nitrate measurements are summarized in Table S1. The initial cultivation summaries include low-salinity observations through day 40 and high-salinity observations through day 57 for STR and day 53 for SUG; later low-salinity follow-up records were not included in these summaries.

The two carbon–nitrogen regimens compared here represent two strategies currently applied in commercial biofloc systems: a low-cost agricultural byproduct (rice straw) combined with an inorganic nitrogen source and a *Lactobacillus* starter, and a conventional regimen based on brown sugar, shrimp feed and a *Pediococcus* starter. Each starter is matched to its substrate: the *Lactobacillus*-based starter is the formulation used with lignocellulose-rich substrates such as rice straw, whereas the *Pediococcus*-based starter is used with readily fermentable sugar substrates. Because the two regimens differ simultaneously in carbon source, nitrogen form and starter culture, this comparison evaluates the overall performance of the two practical strategies rather than the isolated effect of any single factor. Separating the contribution of each factor would require additional factorial treatments and is acknowledged as a limitation of the present design.

### 2.3. Salinity Stress Test of Low-Salinity Mature Bioflocs

Mature bioflocs cultured at low salinity (5 ppt) using rice straw and ammonium chloride were selected based on prior experimental results. This experiment aimed to assess their nitrite degradation efficiency and response to salinity shifts at 15 ppt and 25 ppt. The experiment included four treatment groups, each replicated in triplicate: STR_I: 15 ppt, inoculated with 1 mL/L mature bioflocs; STR_II: 25 ppt, inoculated with 1 mL/L mature bioflocs; CON_I: 15 ppt, supplemented with 100 mg/L sterile rice straw powder; and CON_II: 25 ppt, supplemented with 100 mg/L sterile rice straw powder. Inoculation doses are expressed as wet settled volume (biofloc volume, BFV), i.e., milliliters of settled biofloc per liter of culture water (*v*/*v*), and not as total suspended solids (TSSs, mg/L).

Before the experiment, nitrite nitrogen concentrations in all groups were adjusted to 20 mg/L, with an additional 10 mg/L added on day 7. During the experiment, water samples were collected daily at 9:00 a.m. to measure nitrite nitrogen and total alkalinity. Simultaneously, pH, temperature, and dissolved oxygen were monitored using DINGHAI portable sensors (Guangzhou Dinghai Technology Co., Ltd., Guangzhou, China).

### 2.4. Effect of Bioflocs on the Survival Rate of Diseased Pacific White Shrimp

Pacific white shrimp were reared in three 500 L canvas tanks (100 × 100 × 50 cm) under a biofloc system without water exchange. The C/N ratio was maintained at 15 with a salinity of 5 ppt. During the 37-day rearing period, shrimp exhibited symptoms of lethargy, empty gut, and gradual mortality syndrome. Diagnostic tests confirmed *Vibrio* infections in the hepatopancreas, gut, and muscle tissues. At the start of the experiment, shrimp weighed (0.67 ± 0.002) g.

The formal experiment was also conducted in 12 cylindrical tanks (50 L, 40 cm diameter × 37 cm height), each containing 40 L of water with a salinity of 5 ppt. The experiment consisted of three treatment groups, each with three replicates. The CON group used the original culture water without biofloc addition. The BIO I group used the original culture water inoculated with 1 mL/L mature bioflocs at the start of the experiment. The BIO II group used the original culture water inoculated with 1 mL/L mature bioflocs, but the experiment commenced only after nitrite nitrogen levels in the water decreased to a low level.

During the experiment, water samples were collected daily at 9:00 a.m. to measure ammonia nitrogen and nitrite nitrogen levels in the aquaculture water. Shrimp were fed a commercial diet three times daily (8:00, 14:00, and 20:00) with feeding rates adjusted according to body weight. Continuous aeration was provided 24 h per day, and no water exchange was performed. Survival rates were monitored regularly, and the experiment was terminated on day 24 when significant differences in survival rates were observed among groups. At the conclusion of the experiment, all shrimp in the CON and BIO I groups had died; therefore, only the BIO II group was sampled. Thiosulfate–citrate–bile salts–sucrose (TCBS) agar colony counts were performed on the aquaculture water collected before and after the experiment, as well as on the hepatopancreas, gut, and muscle tissues of shrimp from the BIO II group.

### 2.5. Effect of Bioflocs on the Resistance of Pacific White Shrimp to V. harveyi Infection

To evaluate the potential of bioflocs in enhancing the resistance of Pacific white shrimp to *V. harveyi* infection, this experiment investigated the survival rate of shrimp under controlled conditions with and without biofloc and *V. harveyi* addition. The experiment was conducted using 12 cylindrical tanks (50 L, 40 cm diameter × 37 cm height), each containing 40 L of water with a salinity of 5 ppt. Prior to the experiment, Pacific white shrimp were acclimated in water with a salinity of 5 ppt for 20 days. During acclimation, ammonia nitrogen and nitrite nitrogen levels were maintained at low concentrations. To ensure the shrimp were free of *Vibrio* infection, the gut, hepatopancreas, and muscle tissues of the shrimp were examined prior to the experiment. At the start of the experiment, the shrimp had an average body weight of (1.14 ± 0.07) g.

The experiment included four treatment groups, each with 30 Pacific white shrimp: NN (no biofloc inoculation and no *V. harveyi* strain 1 inoculation), NV (no biofloc inoculation and *V. harveyi* strain 1 inoculation), BN (biofloc inoculation and no *V. harveyi* strain 1 inoculation), and BV (biofloc inoculation and *V. harveyi* strain 1 inoculation). In the NN and NV groups, brown sugar was added daily based on feeding levels to maintain a C/N ratio of 15, and *Bacillus* probiotics were introduced following standard production practices. In the NV and BV groups, *V. harveyi* strain 1 was added every three days at a concentration of 1 × 10^5^ cells/mL. The culture and preparation of the *V. harveyi* strain 1 followed published protocols from our laboratory [15]. In the BN and BV groups, mature bioflocs were inoculated at a concentration of 1 mL/L.

During the experiment, water samples were collected daily at 9:00 a.m. to measure ammonia nitrogen and nitrite nitrogen levels in the aquaculture system. Pacific white shrimp were fed a formulated diet twice daily (8:00 and 17:00), with feeding amounts adjusted based on body weight to ensure adequate nutrition. Continuous aeration was provided 24 h per day, and no water exchange was performed. Survival rates of shrimp in each group were recorded daily, and the experiment was terminated on day 14 when significant differences in survival rates were observed among the groups.

At the beginning and end of the experiment, water and shrimp tissue samples were collected from each group. Water and tissue samples were analyzed for microbial community composition using 16S rDNA amplicon sequencing and for *Vibrio* quantification using TCBS agar colony counting. Additionally, histological analysis of shrimp muscle tissues was performed using hematoxylin and eosin (H&E) staining to assess structural changes.

### 2.6. Water Quality Monitoring

Water samples were collected daily at 9:00 a.m. for water quality monitoring. Ammonia nitrogen, nitrite nitrogen, and total alkalinity were measured using a Dinghai automatic water quality analyzer (Guangzhou Dinghai Technology Co., Ltd., Guangzhou, China). Ammonia nitrogen was determined based on the phenol-indophenol blue method, nitrite nitrogen was measured using the hydrochloric acid–naphthylethylenediamine colorimetric method, and total alkalinity was assessed via acid–base titration. pH, temperature, and dissolved oxygen were monitored in situ using a Dinghai portable pH and dissolved oxygen meter (Guangzhou Dinghai Technology Co., Ltd., Guangzhou, China).

### 2.7. 16S rDNA Analysis of Water and Tissue Samples

During the experiment, 100 mL of water was collected at each sampling point and filtered through a 0.22 μm sterile microporous membrane. The filtered membranes were placed in 5 mL sterile cryotubes and immediately stored at −80 °C for subsequent analysis. For shrimp samples, *P. vannamei* were weighed and surface-disinfected with 75% ethanol. Under sterile conditions, the hepatopancreas and gut were dissected and placed in separate 5 mL sterile cryotubes. The samples were rapidly frozen in liquid nitrogen and stored at −80 °C until further processing.

At the end of the experiment, the stored water sample membranes and shrimp tissue samples were sent to Shanghai Majorbio Bio-Pharm Technology Co., Ltd., Shanghai, China, for high-throughput sequencing. The company performed DNA extraction, PCR amplification of the 16S rDNA (with 0.2 μL BSA included in each 20 μL reaction system to enhance amplification efficiency), Illumina MiSeq sequencing, and subsequent data analysis.

### 2.8. Enumeration of Vibrio Colonies on TCBS Agar

To determine the density of bacteria capable of growing on TCBS agar (hereafter referred to as “TCBS bacteria”) in the hepatopancreas, muscle, gut, and aquaculture water, samples were processed as follows. Tissue samples (hepatopancreas, muscle, and gut) were weighed and homogenized in sterile recirculating aquaculture system (RAS) water at a ratio of 1 g tissue per 1000 mL sterile RAS water to create a homogenate, which was then serially diluted. Water samples from the aquaculture system were directly subjected to serial dilution with sterile RAS water. A 5 μL aliquot of each dilution was spot-inoculated onto TCBS agar plates, with three replicates per sample. After inoculation, the plates were incubated at 28 °C for 18 h in a biochemical incubator. Colony numbers were recorded, and the density of TCBS bacteria was calculated.

### 2.9. Histological Observation of the Muscle of P. vannamei

Muscle samples from Pacific white shrimp were immediately placed in Bouin’s solution for fixation at room temperature. The fixative was replaced with fresh Bouin’s solution the following day to ensure optimal fixation. Samples were fixed for a total of 5 days to preserve tissue integrity and enhance staining quality. Following fixation, the samples were sent to Servicebio Biotechnology Co., Ltd. (Wuhan, China) for paraffin embedding, H&E staining, and microscopic imaging. The preparation of tissue sections and microscopic photography were performed according to Servicebio’s standardized internal protocols. Histological sections were examined under a light microscope to evaluate the structural details of the stained muscle tissues.

### 2.10. Statistical Analyses

**Figure 1:**
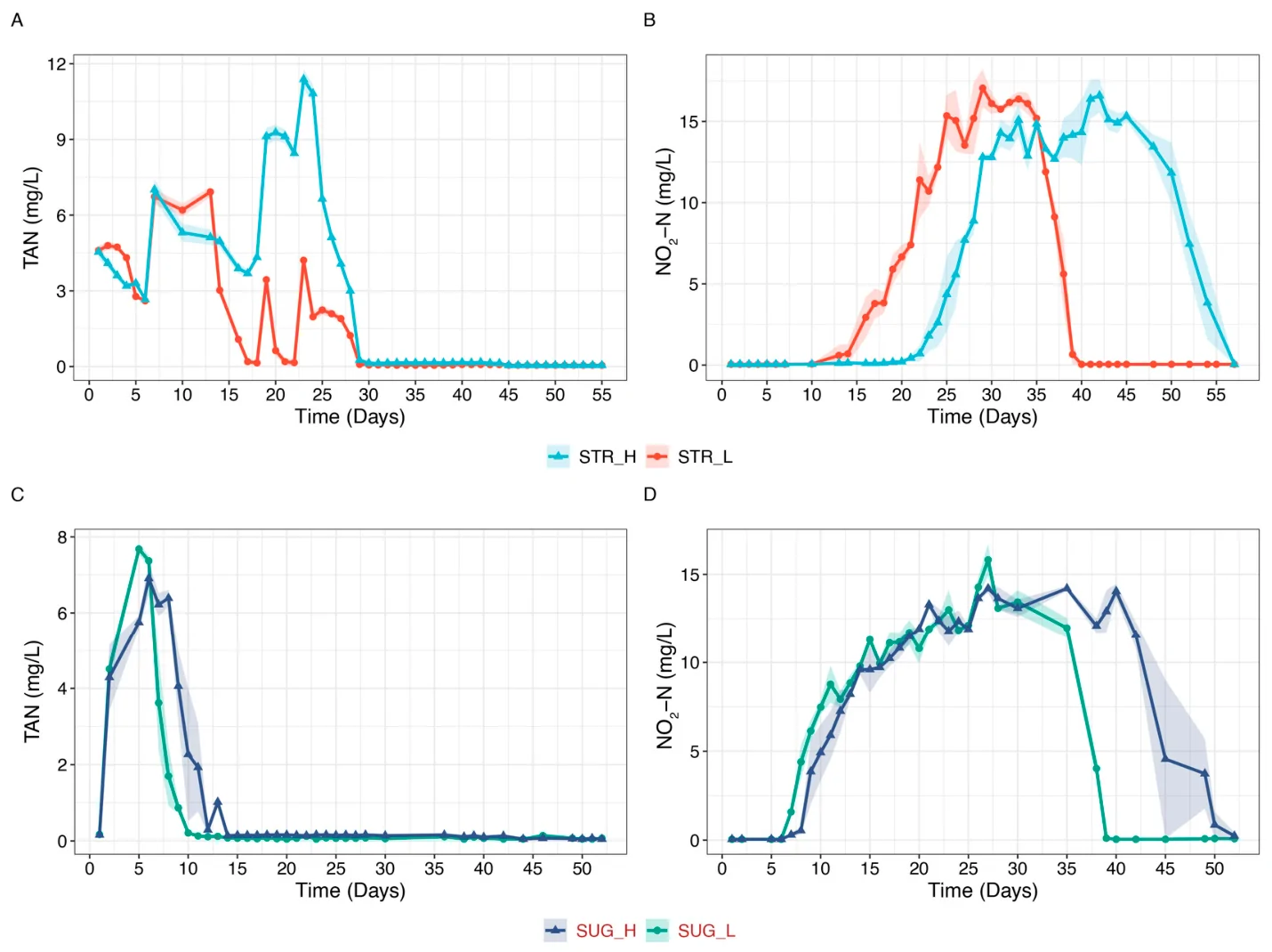
TAN and nitrite nitrogen concentrations during biofloc cultivation. (**A**,**B**) Rice-straw-based systems (STR). (**C**,**D**) Brown-sugar-based systems (SUG). L and H denote salinities of 5 and 25 ppt, respectively. Points represent means and shaded areas indicate ± SE (*n* = 3, except SUG_H on day 47, *n* = 2). Lines connect available measurements as a visual guide. Day 1 denotes the start of each cultivation experiment; the reporting windows are described in Section 2.2.

All raw data were compiled using Microsoft Excel (version 16.0). Statistical analyses were performed using SPSS (version 25.0), and figures were generated using GraphPad Prism (version 9.1) and R software (version 4.5.2). Prior to analysis, data normality and homogeneity of variances were assessed using the Shapiro–Wilk and Levene’s tests. One-way ANOVA was used to evaluate differences among treatment groups, while two-way ANOVA was applied to examine the interaction effects between two independent variables. Significance was determined using Tukey’s HSD test with a threshold set at *p* < 0.05. Results are presented as mean ± standard error (Mean ± SE), with error bars derived from triplicate measurements. Day-14 survival percentages were calculated separately for each independent rearing tank. Differences among the four treatment groups were assessed using one-way ANOVA followed by Tukey’s HSD test. The survival analysis wasgenerated in R version 4.5.2. Statistical significance is indicated by different lowercase letters or asterisks, as specified in each figure legend. Figure 1 summaries were calculated from the available tank measurements, with *n* = 3 except for SUG_H on day 47 (*n* = 2). The initial STR nitrate measurements had *n* = 1 and are reported without SE. Four nitrate group/day records with unresolved source inconsistencies (STR_H on days 21 and 37; SUG_H on days 14 and 27) and negative nitrate estimates in SUG_H on day 40 were excluded from the nitrate summaries. The original records were retained for traceability. Nitrate concentrations were summarized descriptively because the reported treatment end-points occurred on different cultivation days.

## 3. Results

### 3.1. Effects of Salinity and Carbon–Nitrogen Source Interactions on Nitrogen Transformation Efficiency and End-Product Accumulation in Biofloc Systems

To evaluate the interactive effects of salinity and C/N sources on nitrogen transformation in biofloc systems, bioflocs were cultivated at a C/N ratio of 15 under two salinity conditions (5 ppt and 25 ppt) and two carbon–nitrogen source combinations: rice straw with ammonium chloride (STR_L and STR_H) and brown sugar with shrimp feed (SUG_L and SUG_H). Dissolved oxygen (~7.2 mg/L) and pH (~8.25) remained stable throughout the experiment, with no significant differences observed between treatments.

In the rice-straw-based systems (Figure 1A,B), TAN declined to low residual concentrations by day 17 in STR_L and day 30 in STR_H. Nitrite nitrogen concentrations were 0.05 mg N/L in STR_L on day 40 and in STR_H on day 57. Nitrate nitrogen concentrations were 8.71 ± 0.26 mg N/L in STR_L on day 40 and 7.51 ± 0.32 mg N/L in STR_H on day 57 (Table S1).

In the brown-sugar-based systems (Figure 1C,D), nitrite nitrogen concentrations declined to 0.11 ± 0.03 mg N/L in SUG_L on day 40 and 0.24 ± 0.19 mg N/L in SUG_H on day 53. Nitrate nitrogen concentrations at these sampling points were 4.96 ± 0.73 and 14.10 ± 0.11 mg N/L, respectively (Table S1). These nitrate values represent measurements on the stated cultivation days rather than a continuous, time-matched series.

Overall, low salinity (5 ppt) consistently accelerated ammonia and nitrite removal in both carbon–nitrogen source combinations, though nitrate accumulation patterns varied depending on salinity and substrate type.

### 3.2. Impact of Salinity on Microbial Community Structure and Diversity in Bioflocs Cultured with Different Carbon and Nitrogen Sources

To assess how salinity influences microbial community structure and diversity in biofloc systems with distinct C/N sources, bioflocs were cultivated under low- (STR_L) and high (STR_H)-salinity conditions using rice straw and ammonium chloride. Nitrite removal was completed by day 40 in the low-salinity group and day 57 in the high-salinity group. Microbial community samples were collected on days 20, 40, and 57. After quality control and filtering, 37,941 high-quality sequences were obtained for analysis. Alpha diversity indices (Table 1) revealed significantly higher richness and diversity in the low-salinity group compared to the high-salinity group (*p* < 0.05). On day 40, the operational taxonomic units (OTUs) in the low-salinity group were 613 ± 3 versus 445 ± 12 in the high-salinity group. Similarly, the Sobs index was higher in the low-salinity group (277.67 ± 5.93) than in the high-salinity group (241.33 ± 8.74) at the point of nitrite depletion (*p* < 0.05), a trend also reflected in the Shannon diversity index.

**Figure 2:**
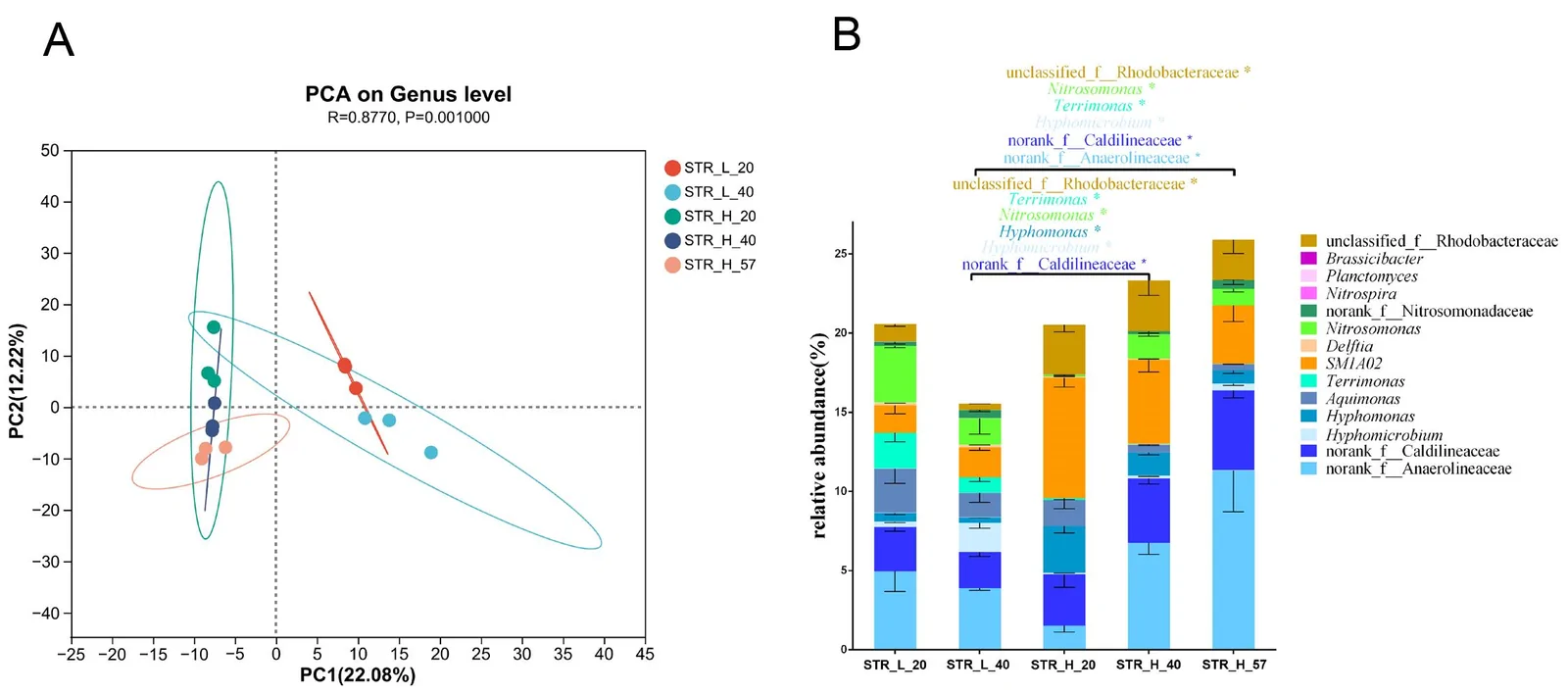
Microbial community composition and structure under different salinity conditions using rice straw and ammonium chloride as carbon and nitrogen sources. (**A**) Principal component analysis (PCA) of microbial communities at the genus level under low-salinity (STR_L) and high-salinity (STR_H) conditions. (**B**) Relative abundances of dominant nitrogen-cycling genera (>1%) at different culture stages. Asterisks indicate significant differences between groups (* *p* < 0.05).

Principal component analysis (PCA) demonstrated a clear separation between the two salinity groups along PC1, explaining 60.8% of the total variation (Figure 2A), indicating that salinity was a primary driver of microbial community composition. At the phylum level, Proteobacteria, Bacteroidetes, Actinobacteria, Chloroflexi, and Planctomycetes dominated (>1% relative abundance) in both groups (Table S2). Although nitrogen-cycling genera were detected in both conditions (Figure S1), their relative abundances differed (Figure 2B). SM1A02 was significantly enriched in the high-salinity group, while *Delftia* was moderately higher in the low-salinity group (not statistically significant). On day 20, ammonia-oxidizing bacteria (e.g., *Nitrosomonas* and norank_f_Nitrosomonadaceae) were more abundant in the low-salinity group but declined by later stages. Nitrifiers remained below 0.05% in both groups, and *Nitrospira* was undetected under high salinity. Denitrifying bacteria (e.g., norank_f_Caldilineaceae) showed higher abundance in the high-salinity group, whereas *Hyphomicrobium* and *Aquimonas* were more prevalent under low salinity. Heterotrophic bacteria (unclassified_f_Rhodobacteraceae) were consistently more abundant in the high-salinity group.

Similarly, microbial communities in bioflocs cultivated with brown sugar and shrimp feed (SUG_L and SUG_H) were analyzed on days 40 and 53. Alpha diversity indices (Chao1 and Sobs) were significantly higher under low salinity (*p* < 0.05), while the Shannon index exhibited a nonsignificant upward trend (Table S3). PCA (Figure S2A) showed distinct separation along PC1, consistent with earlier findings. At the phylum level, SUG_L exhibited broader phylum diversity (Table S4). Nitrogen-cycling genera analysis (Figure S2B,C) revealed higher SM1A02 in SUG_H and elevated *Delftia* in SUG_L, though differences in ammonia-oxidizing bacteria were not significant. Denitrifiers (e.g., norank_f_Caldilineaceae and norank_o_JG30-KF-CM45) were more prevalent in the high-salinity group, while unclassified_f_Rhodobacteraceae reached 2.05% in SUG_H compared to 0.12% in SUG_L (*p* < 0.05).

Overall, low-salinity conditions (5 ppt) significantly enhanced microbial richness and diversity under both carbon–nitrogen source treatments. Although SUG showed slightly higher microbial richness at low salinity (Table S3), both groups achieved complete nitrite removal by day 40. From an economic perspective, STR offers greater advantages. As a low-cost and readily available agricultural byproduct, rice straw helps reduce production costs and improve resource utilization, making it a more suitable option for large-scale aquaculture applications. Taken together, the STR group under low salinity (5 ppt) exhibited the best overall performance in nitrogen cycling efficiency, microbial diversity, and cost-effectiveness, highlighting its strong potential for practical applications.

### 3.3. NO_2_^−^-N Removal Efficiency of Mature Bioflocs Under Varying Salinity Conditions

**Figure 3:**
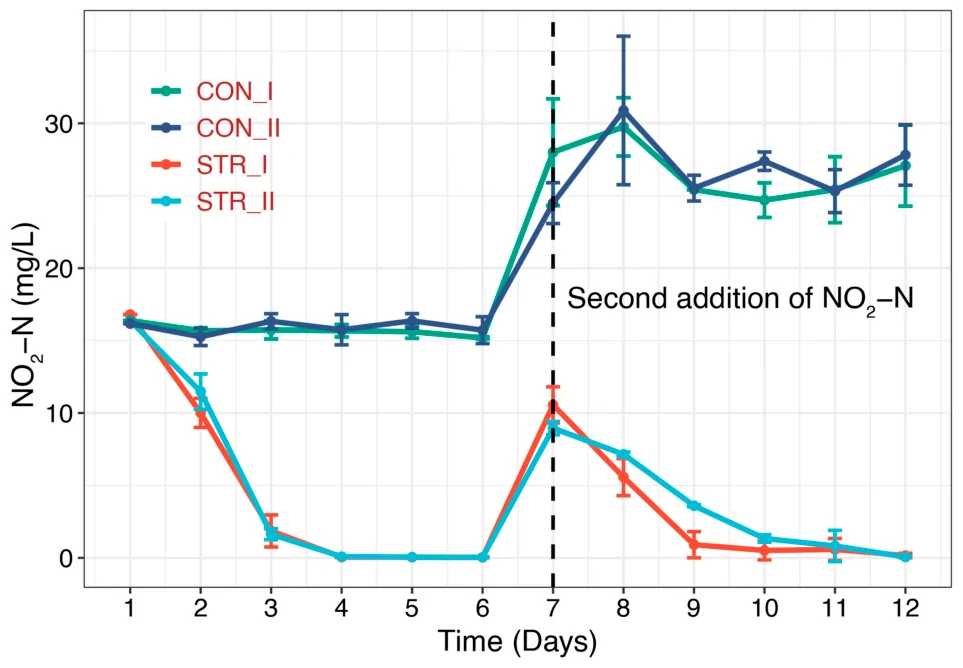
Temporal dynamics of nitrite nitrogen (NO_2_^−^-N) concentrations during removal by mature bioflocs under varying salinity conditions. Mature bioflocs cultivated under low salinity (STR_L) were inoculated into systems with moderate (15 ppt, STR_I) and high salinity (25 ppt, STR_II). Control groups (CON_I and CON_II) received no biofloc inoculation. The vertical dashed line at day 7 indicates the second addition of NO_2_^−^-N. Data are presented as mean ± standard error (SE, *n* = 3).

To assess the adaptability and nitrite removal capacity of mature bioflocs cultivated under low-salinity conditions (STR_L), inoculations were performed at moderate- (15 ppt, STR_I) and high-salinity (25 ppt, STR_II) levels. As shown in Figure 3, both treatment groups achieved complete NO_2_^−^-N removal (0 mg/L) within four days after the first addition. Upon the second addition of NO_2_^−^-N on day 7, STR_I reached 0 mg/L one day earlier than STR_II; however, no statistically significant difference (*p* > 0.05) was observed in the overall removal efficiency between the two groups. In contrast, the control groups (CON_I and CON_II) exhibited negligible NO_2_^−^-N removal throughout the experiment. These results highlight that mature bioflocs maintain robust NO_2_^−^-N removal performance under both moderate and high-salinity conditions, with salinity exerting minimal influence on overall nitrogen transformation kinetics.

### 3.4. Biofloc Application Improves Water Quality and Survival of Vibrio-Infected P. vannamei

**Figure 4:**
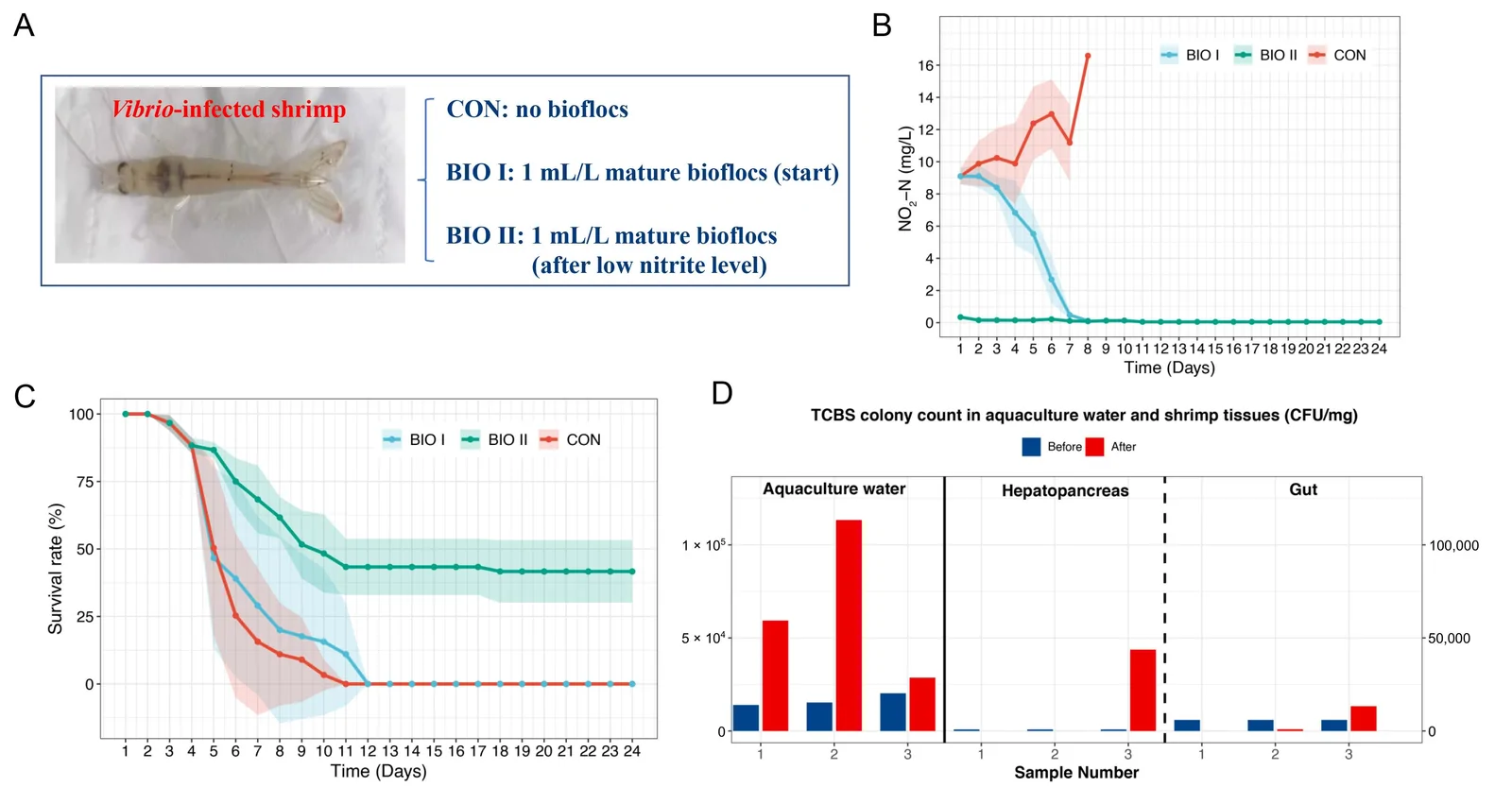
Effects of mature biofloc inoculation on water quality, shrimp survival, and *Vibrio* load in *Vibrio*-infected *P. vannamei*. (**A**) Experimental design. Schematic representation of the three treatment groups in the *Vibrio*-infected *P. vannamei* culture: a control group without bioflocs (CON), a group with mature biofloc inoculation at the experiment’s start (BIO I), and a group with mature biofloc inoculation after nitrite levels decreased (BIO II). (**B**) Nitrite–nitrogen (NO_2_^−^-N) concentrations over time. Time-course measurements of NO_2_^−^-N concentrations in rearing water across treatments. Shaded areas indicate standard deviations. (**C**) Survival curves of *P. vannamei*. Survival rates (%) of shrimp under the three treatments over the 24-day trial. (**D**) *Vibrio* loads in aquaculture water and shrimp tissues (hepatopancreas and gut). Colony-forming units (CFU mg^−1^) on TCBS agar measured before and after the trial.

Mature bioflocs cultivated at 5 ppt using rice straw and ammonium chloride were evaluated for their effects on water quality and the survival of *Vibrio*-infected *P. vannamei* (Figure 4A). Ammonia–nitrogen levels remained low (0–0.1 mg/L) in all groups, while nitrite–nitrogen levels (Figure 4B) steadily increased in the control (CON), exceeding 10 mg/L by the end of the experiment. The BIO I group showed a decline in nitrite to 0 mg/L by day 8, whereas the BIO II group maintained consistently low concentrations throughout.

Survival curves of *P. vannamei* (Figure 4C) revealed complete mortality in the CON group by day 11 and in the BIO I group by day 12. In contrast, shrimp in the BIO II group maintained a survival rate of approximately 41% through day 24, significantly higher than those in the other groups (*p* < 0.05). These results suggest that pre-incubated bioflocs provide substantial survival benefits by stabilizing water quality and mitigating pathogen impacts during *Vibrio* exposure.

TCBS agar counts from the BIO II group (Figure 4D) indicated elevated bacterial loads in the water column but notably lower colony counts in the hepatopancreas and gut of shrimps at the trial’s conclusion. This shift suggests that pre-incubated bioflocs promote beneficial microbial communities, potentially limiting TCBS-culturable bacterial counts within host tissues. Variations in gut bacterial loads among replicates may reflect differences in individual physiology or pathogen susceptibility.

### 3.5. Biofloc Inoculation Significantly Mitigates Vibrio harveyi Infection and Enhances P. vannamei Survival

**Figure 5:**
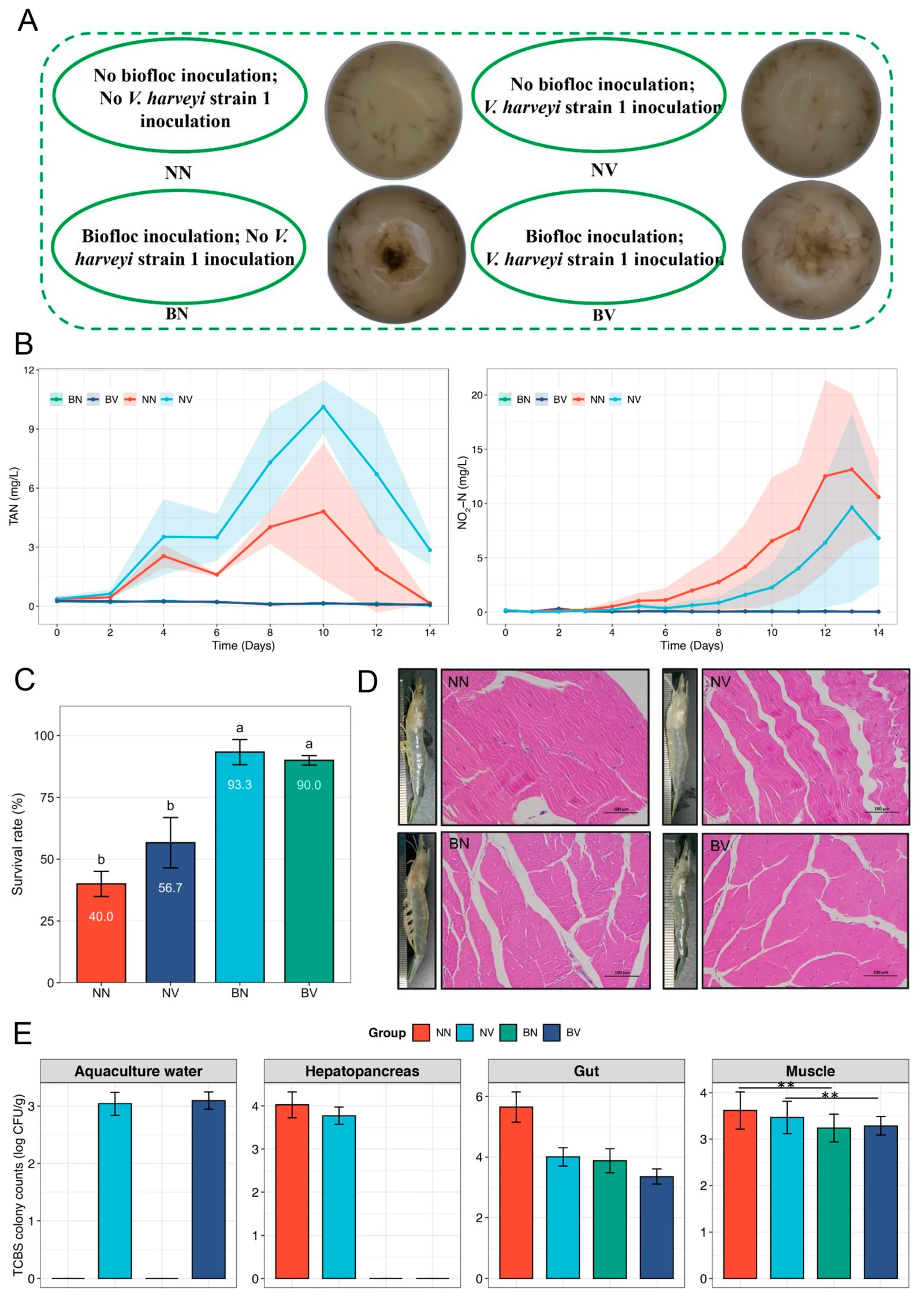
Impact of biofloc inoculation on water quality, survival, and *Vibrio harveyi* infection in *P. vannamei*. (**A**) Overview of experimental treatments. Four groups were tested: NN (no biofloc, no *V. harveyi*), NV (no biofloc, *V. harveyi*), BN (biofloc), and BV (biofloc + *V. harveyi*). Representative images of shrimp appearance are shown. (**B**) Changes in total ammonia–nitrogen (TAN) and nitrite–nitrogen (NO_2_^−^-N) concentrations over the 14-day trial. Shaded areas represent standard deviations. (**C**) Final shrimp survival rates on day 14. Bars represent mean ± SE (*n* = 3 independent rearing tanks per treatment). Different lowercase letters indicate significant differences among groups (one-way ANOVA followed by Tukey’s HSD test, *p* < 0.05). (**D**) Hematoxylin and eosin (H&E) stained muscle tissue sections of *P. vannamei* from each treatment group. (**E**) *Vibrio* loads (log CFU/g) in aquaculture water and shrimp tissues (hepatopancreas, gut, and muscle), pre- and post-challenge, determined by TCBS agar enumeration. Asterisks indicate significant differences between groups (** *p* < 0.01).

The highly virulent *V. harveyi* strain 1 was used to infect shrimp under four treatments: NN and NV (without biofloc) and BN and BV (with biofloc) (Figure 5A). This pathogen is known to cause severe muscle lesions in *P. vannamei*. During the 14-day trial, BN and BV consistently maintained low ammonia and nitrite levels. In contrast, NN and NV exhibited elevated ammonia concentrations, with NV surpassing NN. Conversely, NN had significantly higher nitrite concentrations compared to NV (Figure 5B). While nitrate accumulation differed across treatments, these variations did not reach statistical significance.

Day-14 survival was significantly higher in BN (93.3%) and BV (90.0%) than in both NN (40.0%) and NV (56.7%) (Tukey-adjusted *p* < 0.05; Figure 5C). No significant differences were detected between NN and NV or between BN and BV (Tukey-adjusted *p* > 0.05). No significant interaction between biofloc addition and *Vibrio* exposure was detected (two-way ANOVA, *p* = 0.152).

Histological examination (Figure 5D) showed pronounced muscle necrosis and fiber disruption in the NN and NV groups, whereas BN and BV maintained intact muscle architecture. TCBS agar enumeration (Figure 5E) revealed no detectable *Vibrio* colonies in the water of NN and BN groups. In contrast, NV exhibited higher *Vibrio* loads than BV. Consistently, TCBS-culturable bacterial counts in the gut and muscles were elevated in NN and NV, whereas BN and BV displayed markedly lower levels. These findings demonstrate that biofloc inoculation effectively mitigates *V. harveyi* infection, enhances shrimp survival, and promotes microbial community shifts that reduce pathogen colonization.

### 3.6. Dual-Factor Effects of Biofloc Inoculation and Vibrio harveyi Challenge on Water and Shrimp Tissue Microbiota

High-throughput 16S rRNA gene sequencing was conducted to evaluate the microbial communities in water and shrimp tissues (gut, hepatopancreas, and muscle) under dual-factor treatments involving biofloc inoculation and *V. harveyi* challenge.

**Figure 6:**
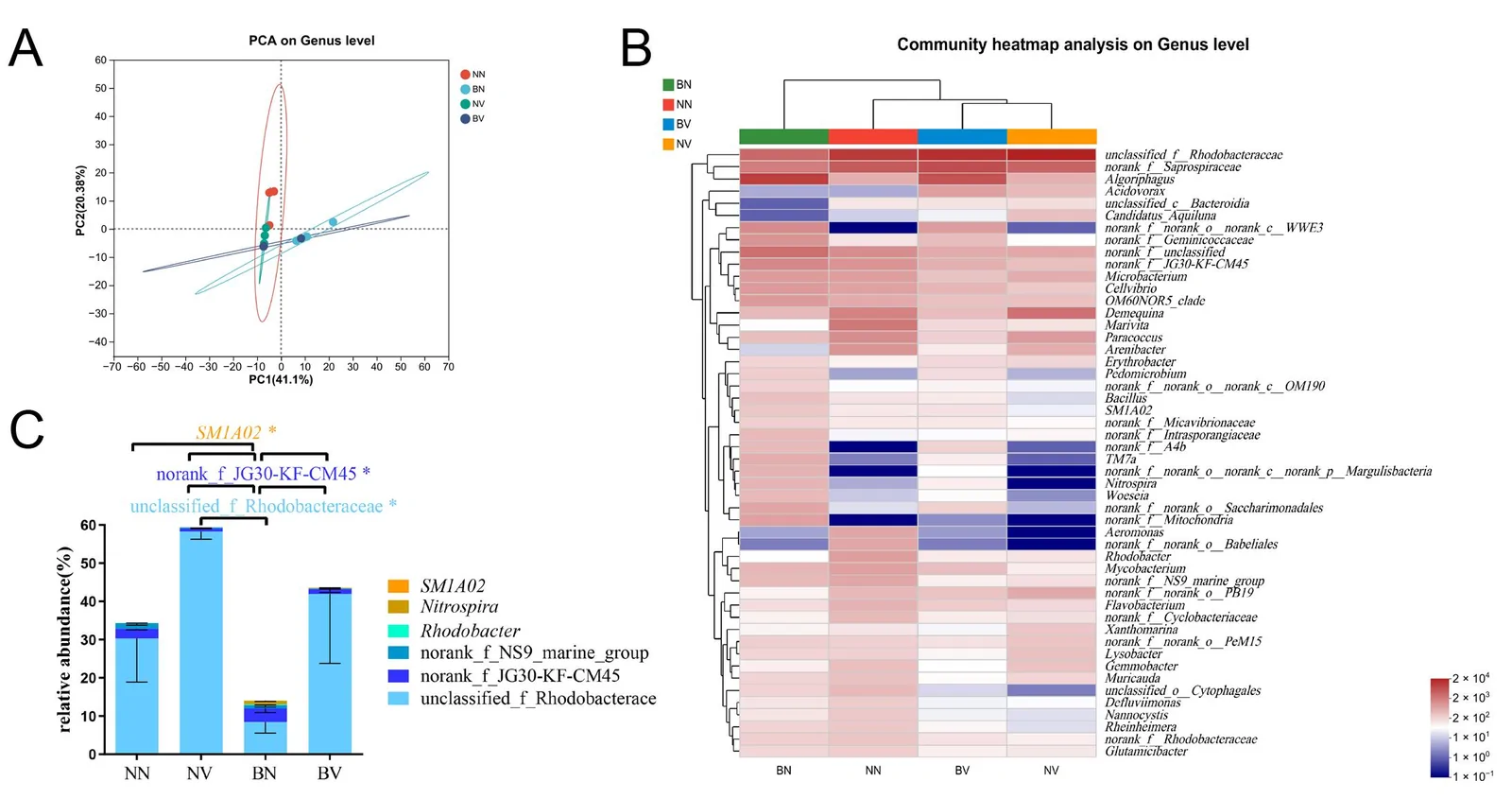
Effects of biofloc inoculation and *Vibrio harveyi* challenge on microbial communities in aquaculture water. (**A**) Principal component analysis (PCA) of genus-level microbial communities. Each point represents an individual sample, with ellipses indicating 95% confidence intervals. (**B**) Heatmap of the top 35 genera across treatments. Color intensity reflects log10-transformed relative abundances. (**C**) Relative abundances of nitrogen-cycling bacteria. Bars show mean ± standard deviation (SD). Asterisks indicate significant differences among treatments (one-way ANOVA with Tukey’s HSD test, * *p* < 0.05).

In the water column (Table 2), alpha diversity analysis revealed that the BN group exhibited significantly higher Chao1, Sobs, and Shannon indices compared to the NV and BV groups (*p* < 0.05), indicating that biofloc inoculation enhanced microbial richness and diversity. Conversely, the NV and BV groups showed significantly higher Simpson indices than NN and BN (*p* < 0.05), suggesting reduced evenness following *Vibrio* exposure. No significant interaction was observed between biofloc inoculation and *Vibrio* challenge regarding microbial diversity metrics. Principal component analysis (PCA) revealed distinct microbial clustering patterns (Figure 6A). The NN and NV groups clustered closely, indicating similar microbial compositions, while BV partially overlapped with both NV and BN. In contrast, BN displayed a distinct microbial community structure (PC1: 41.1%; PC2: 20.38%). At the phylum level, Proteobacteria and Bacteroidetes were dominant across all groups. NN primarily consisted of Proteobacteria (58.29%), Bacteroidetes (24.58%), and Actinobacteria (9.46%). NV showed an increase in Proteobacteria (69.52%) with a reduction in Bacteroidetes (16.33%). In the BN group, Proteobacteria (38.08%) and Bacteroidetes (36.36%) were nearly balanced, accompanied by an elevated abundance of Patescibacteria (6.20%). BV was similarly dominated by Proteobacteria (59.89%) and Bacteroidetes (35.29%). Genus-level heatmap analysis (Figure 6B) revealed notable shifts in microbial composition. Unclassified_f_Rhodobacteraceae was the most abundant genus in NN, NV, and BV (30.57%, 58.46%, and 42.11%, respectively), while norank_f_Saprospiraceae displayed similar patterns (12.66%, 9.67%, and 17.21%). In contrast, BN was enriched with *Algoriphagus* (26.73%), with a markedly reduced abundance of unclassified_f_Rhodobacteraceae (8.5%). Notably (Figure 6C), *Algoriphagus* was also prevalent in BV (15.39%). *Vibrio* inoculation significantly increased unclassified_f_Rhodobacteraceae abundance in NV (*p* < 0.05), while reducing the relative abundances of norank_f_JG30-KF-CM45, *Nitrospira*, and SM1A02 compared to BN. Elevated abundances of *Nitrospira* and SM1A02 in BN suggest enhanced nitrification potential in biofloc-treated systems.

In the shrimp gut, the NV and BV groups exhibited significantly higher Chao1, Sobs, and Shannon indices than NN and BN (*p* < 0.05), indicating increased microbial richness following *Vibrio* exposure (Table S5). PCA (PC1: 48.06%; PC2: 9.86%) revealed two distinct clusters, with NN and NV forming one cluster and BN and BV forming another (Figure S3A). At the genus level (Figure S3B), *Paracoccus* (17.90%, 26.72%) and *Demequina* (15.02%, 11.58%) dominated NN and NV, whereas BN and BV were enriched with norank_f_JG30-KF-CM45, *Paracoccus*, and *Bacillus*. Notably, *Bacillus* was significantly more abundant in BN compared to NN and NV (*p* < 0.05). Sequences affiliated with the genus *Vibrio* were not recovered from the gut of NV, BN, or BV, and were detected only in NN (Figure S3C).

In the hepatopancreas, NN and NV samples were dominated by Proteobacteria and Actinobacteria, while BN and BV showed higher proportions of Patescibacteria. Notably, TM7a and norank_f_Saccharimonadales were significantly more abundant in BN and BV than in NN and NV (*p* < 0.05). Sequences affiliated with the genus *Vibrio* were detected only in NN samples (Figure S4A,B).

Muscle tissue microbiota revealed distinct genus-level profiles. NN was enriched with *Achromobacter* (17.58%), *Bacillus* (10.16%), and *Shewanella* (20.15%). NV was characterized by higher relative abundances of unclassified_f_Rhodobacteraceae (18.25%) and *Paracoccus* (14.31%) (Figure S4C). In contrast, BN and BV were predominantly composed of *Achromobacter* and *Bacillus*, with biofloc inoculation significantly increasing *Bacillus* abundance (*p* < 0.05). Sequences affiliated with the genus *Vibrio* were detected only in NN and NV and were not recovered from BN or BV (Figure S4D).

## 4. Discussion

### 4.1. Low Salinity Accelerates Ammonia and Nitrite Removal and Enriches Key Nitrogen-Cycling Bacteria in Bioflocs

This study explored how salinity (5 ppt vs. 25 ppt) and carbon–nitrogen sources influence nitrogen removal efficiency and microbial community dynamics in biofloc systems. Salinity is a key factor affecting biofloc maturation, with higher levels inhibiting nitrification [35]. Under low-salinity conditions, TAN declined to low residual concentrations by day 17 and nitrite nitrogen by day 40, whereas high salinity extended these times to approximately 30 days for TAN and 57 days for nitrite. These findings suggest that elevated salinity impairs microbial activity involved in nitrogen cycling and consequently slows the transformation processes.

High-throughput sequencing revealed a distinct succession of functional microbial groups under low-salinity conditions. Ammonia-oxidizing bacteria (AOB), including *Nitrosomonas* and norank_f_Nitrosomonadaceae, were dominated by the conversion of NH_4_^+^ to NO_2_^−^. Later stages involved facultative denitrifiers (e.g., *Hyphomicrobium*, *Aquimonas*) and Actinobacteria, which facilitated NO_2_^−^ reduction to N_2_ gas and contributed to organic matter degradation [36,37]. The low-salinity environment likely enhances microbial metabolism and diversity, promoting efficient coupling of nitrification and denitrification and thus preventing nitrite accumulation [38]. In contrast, high salinity enriched halotolerant taxa such as SM1A02 and unclassified_f_Rhodobacteraceae. However, overall microbial diversity and functional versatility declined. Elevated salinity induces osmotic stress, forcing nitrifiers to divert energy from nitrogen metabolism to maintain cellular homeostasis [39]. This metabolic trade-off, combined with the suppression of non-halotolerant heterotrophs and Actinobacteria, impaired both nitrification and denitrification, extending nitrogen removal times and promoting nitrate accumulation [40,41].

Carbon–nitrogen source selection significantly influenced nitrogen dynamics. The SUG_L treatment enabled rapid heterotrophic nitrogen uptake, reaching a lower nitrate nitrogen concentration on day 40 (4.96 ± 0.73 mg N/L) than SUG_H on day 53 (14.10 ± 0.11 mg N/L), but it required frequent carbon additions. Conversely, STR_L provided a sustained carbon source through gradual cellulose degradation. Despite a slower initial removal rate, STR_L and SUG_L both reduced nitrite nitrogen to low residual concentrations by day 40 under low salinity. Notably, STR_L achieved this without continuous carbon supplementation, highlighting its operational simplicity and cost efficiency. Moreover, STR_L supported a stable microbial community with Actinobacteria exceeding 10% abundance, which may enhance nitrogen cycling and pathogen suppression [42].

Nitrate was measured intermittently, and the reported treatment end-points occurred on different cultivation days. These observations therefore describe nitrate concentrations at selected sampling points and do not resolve a continuous, time-matched nitrate trajectory. Moreover, TAN, nitrite, and nitrate concentration measurements alone do not establish a complete nitrogen mass balance, which requires accounting for nitrogen inputs, outputs, and storage in the relevant dissolved, particulate, and biomass pools. The present results should therefore be interpreted as changes in measured aqueous inorganic nitrogen concentrations rather than quantitative partitioning of nitrogen among transformation pathways. Ammonia volatilization was not quantified in this experiment: no gaseous ammonia emission measurements were available, so the contribution of physical ammonia stripping to the observed decline in aqueous TAN under continuous aeration cannot be determined. The decreases in measured TAN reported here should therefore not be attributed exclusively to microbial nitrogen transformation, and the present data are not sufficient to establish a complete nitrogen budget for the system.

In summary, low salinity (5 ppt) enhances nitrogen removal and promotes stable, diverse microbial communities in biofloc systems. Among the treatments, rice straw with ammonium chloride under low salinity offers a superior balance of efficiency, microbial resilience, and economic viability. Its gradual carbon release minimizes supplementation needs, lowers costs, and maintains functional microbial ecosystems, making it a practical solution for high-density shrimp farming [43].

### 4.2. Stable Nitrite Removal by Mature Bioflocs Under Varying Salinity Conditions

NO_2_^−^-N is a highly toxic nitrogenous compound in shrimp aquaculture. Even at low concentrations, it can impair feed intake, inhibit growth, suppress immunity, and increase vulnerability to bacterial infections [24]. Meanwhile, euryhaline shrimp exhibit notable sensitivity to salinity fluctuations [44,45]. Therefore, the ability of bioflocs to maintain stable nitrite conversion under changing salinity conditions is crucial for system resilience and production safety.

Mature bioflocs cultivated at 5 ppt with rice straw and ammonium chloride exhibited stable NO_2_^−^-N removal when transferred to 15 ppt (STR_I) and 25 ppt (STR_II). In both treatments, nitrite concentrations decreased to 0 mg/L within four days, indicating strong salinity tolerance and sustained nitrogen transformation capacity. After a second nitrite addition on day 7, the 15 ppt system completed removal one day earlier than the 25 ppt system, suggesting that moderate salinity slightly accelerates initial degradation rates.

Long-term biofloc cultivation is known to enhance ecological redundancy and microbial stability [36,46], enabling core nitrogen-cycling microorganisms to remain functional across diverse salinity conditions. Consistent with these findings, our results demonstrate that mature bioflocs established at low salinity retained their nitrite-removal capacity following sudden salinity increases. The use of pre-established mature bioflocs enabled complete nitrite removal after the salinity increase, thereby mitigating nitrogen stress in shrimp farming operations. Cell lysis, floc size distribution and total organic carbon were not determined in this experiment, so the functional evidence presented here provides only indirect support for the resilience of the biofloc community.

Although recognized autotrophic nitrifiers such as *Nitrosomonas* and *Nitrospira* accounted for less than 0.05% of the community, nitrite added at 20 mg/L was completely removed within four days. This apparent mismatch suggests that heterotrophic nitrification and aerobic denitrification, rather than autotrophic nitrification, were likely the dominant nitrite-removal pathways in these biofloc systems [41,47,48]. Heterotrophic nitrifiers and aerobic denitrifiers remain active under high organic load and low dissolved oxygen, the conditions that prevailed in the present tanks, which would explain the rapid nitrite depletion despite the low abundance of autotrophic nitrifiers.

### 4.3. Pre-Established Mature Bioflocs Improve Shrimp Survival by Mitigating Nitrogen Stress and Vibrio Colonization

Microbial communities play pivotal roles in aquaculture ecosystems, influencing nutrient cycling and disease resistance. BFT is increasingly recognized as a sustainable approach for improving water quality and controlling infectious diseases in shrimp aquaculture [25]. In this study, practical intensive aquaculture conditions were simulated using canvas tanks without water exchange. Accumulation of organic matter from uneaten feed and feces led to declining DO levels and elevated TAN, both of which are key constraints to intensive shrimp production [49]. Consequently, shrimp exhibited lethargy, empty guts, and high mortality, with *Vibrio* detected in hepatopancreas, gut, and muscle tissues.

In the BIO I treatment, pre-established mature bioflocs were introduced at the experiment’s onset to facilitate nitrogen conversion. However, elevated initial nitrite concentrations exposed shrimp to early water quality stress, increasing susceptibility to *Vibrio* infection. Conversely, in BIO II, mature bioflocs were pre-incubated, and shrimp were stocked only after nitrite levels stabilized at low concentrations, ensuring favorable water conditions. As a result, the BIO II group maintained a survival rate of 41% (*p* < 0.05) by day 24, significantly outperforming both the control (CON) and BIO I groups, which experienced complete mortality. Despite elevated *V. harveyi* loads in the water, colonization within shrimp tissues was notably reduced in BIO II, suggesting effective pathogen exclusion. Beyond microbial competition, bioflocs may also stimulate shrimp immune responses, as indicated in previous studies [50]. These mechanisms promote beneficial microbial communities while suppressing pathogenic taxa, consistent with reports of BFT mitigating diseases like AHPND through competitive exclusion [7,26].

In summary, pre-established mature bioflocs contributed to improved water quality by reducing nitrite accumulation and minimizing TCBS-culturable bacterial counts.

### 4.4. Biofloc Inoculation Enhances Shrimp Survival, Modulates Microbial Communities, and Mitigates Vibrio-Induced Nitrogen Cycle Disruption

This study simulated real-world intensive aquaculture conditions by concurrently introducing *V. harveyi* and mature bioflocs to evaluate their combined effects on shrimp survival, microbial community dynamics, and nitrogen cycling. Such a dual-factor design mirrors the practical scenario where disease outbreaks and water quality deterioration occur simultaneously, enabling a comprehensive analysis of how bioflocs buffer pathogen stress while stabilizing the aquatic environment.

Shrimp in the NN group (no biofloc or *Vibrio* inoculation) exhibited high mortality and severe muscle necrosis, similar to those in the NV group (*Vibrio*-inoculated without bioflocs), highlighting that elevated ammonia and nitrite levels rather than *Vibrio* infection alone were the primary mortality drivers. In contrast, shrimp in the BN and BV groups (biofloc-inoculated) demonstrated significantly improved survival rates, emphasizing the protective role of bioflocs in mitigating both environmental stress and pathogen pressure. Notably, the BN group exhibited the highest survival, suggesting that pre-established bioflocs confer immediate benefits in stabilizing the aquaculture environment. Previous studies have demonstrated that elevated ammonia and nitrite levels can adversely affect the survival of *P. vannamei* [51]. The *Vibrio* challenge notably reduced microbial richness and diversity in rearing water and disrupted biofloc formation. PCA analyses revealed that *Vibrio* exposure led to substantial shifts in microbial communities, particularly decreasing the relative abundances of key autotrophic nitrifiers, including SM1A02 [52] and *Nitrospira* [53], which are essential for classical ammonia-to-nitrite and nitrite-to-nitrate conversion pathways. Although Rhodobacteraceae (facultative photoautotrophs) became more dominant after *Vibrio* inoculation, their reliance on heterotrophic nitrification and aerobic denitrification pathways [47] is less efficient, leading to the temporary retention of nitrogen species (ammonia, nitrite) and water-quality deterioration [48]. These findings underscore how *Vibrio* infection can disrupt nitrogen cycling, compounding environmental stress.

Importantly, biofloc inoculation counteracted these disruptions. Mature bioflocs serve as microbial reservoirs capable of replenishing key nitrifiers and sustaining nitrogen cycling under stress conditions [54]. After biofloc addition, the abundances of *Nitrospira* and norank_f_JG30-KF-CM45 (key nitrifiers) significantly recovered, suggesting that mature bioflocs provide a microbial reservoir that supports nitrogen cycling stability under pathogenic stress. This microbial resilience likely results from the competitive and cooperative interactions within biofloc-associated communities. Additionally, bioflocs are known to interfere with *V. harveyi* quorum-sensing mechanisms [55], potentially reducing *Vibrio* virulence and further supporting microbial community balance. Beyond water column effects, biofloc supplementation enriched the shrimp gut microbiota, enhancing both microbial richness and functional diversity. Biofloc-associated dietary intake improves the functional diversity and stability of the gut microbiota, promoting the enrichment of beneficial bacteria [50]. In the absence of bioflocs, the NN and NV groups exhibited higher relative abundances of potential pathogens such as *Aeromonas* and *Acinetobacter*, which correlated with elevated ammonia and nitrite concentrations. Conversely, the BN and BV groups demonstrated increased *Bacillus* abundance, an established probiotic genus known to enhance host immunity, promote gut health, and improve feed efficiency [56]. *Bacillus* enrichment not only improved microbial community structure but also likely contributed to host resilience through antimicrobial peptide production and modulation of immune-related gene expression [53]. Similar trends were observed in muscle tissues, where biofloc-inoculated groups exhibited significantly reduced pathogenic bacterial loads.

In summary, this study demonstrates that pre-established mature bioflocs can simultaneously improve shrimp survival, stabilize nitrogen cycling, and modulate microbial community structure under dual environmental and pathogenic stress. By enriching beneficial microbiota and suppressing pathogenic bacteria, biofloc technology offers a practical solution for enhancing disease resistance and improving water quality in intensive shrimp aquaculture.

## 5. Conclusions

In conclusion, our findings demonstrate that mature bioflocs established under low-salinity conditions using rice straw and ammonium chloride represent a robust, cost-effective approach to improving water quality and reducing *Vibrio*-related mortality in *P. vannamei* culture. The choice of rice straw, an abundant and inexpensive agricultural byproduct, contributed to a stable and diverse microbial community, expediting ammonia and nitrite removal while maintaining system resilience across salinity gradients. Notably, these mature bioflocs effectively protected shrimp health both when introduced post-infection and more favorably when pre-inoculated prior to stocking, highlighting the preventive potential of well-established microbial consortia. By mitigating nitrogenous wastes and curbing pathogenic *Vibrio* proliferation, this approach offers a promising alternative to traditional water exchange systems, thereby aligning with sustainability goals in high-density aquaculture.

## Figures and Tables

**Table 1:** Richness and diversity indices of microbial communities in water during biofloc cultivation using rice straw and ammonium chloride as carbon and nitrogen sources.

Sample	OTUs	Sobs	Shannon	Simpson	Ace	Chao1
STR_L_20	506 ± 24 a	255.00 ± 2.52 b	4.08 ± 0.08 a	0.03 ± 0.00 b	290.92 ± 5.60	288.38 ± 8.30 ab
STR_L_40	613 ± 3 a	277.67 ± 5.93 a	4.08 ± 0.03 a	0.03 ± 0.00 b	307.35 ± 7.90	318.75 ± 11.7 a
STR_H_20	413 ± 27 b	218.33 ± 5.17 c	3.71 ± 0.03 b	0.04 ± 0.00 a	272.62 ± 17.00	267.27 ± 3.75 c
STR_H_40	445 ± 12 b	241.33 ± 8.74 bc	3.85 ± 0.04 b	0.04 ± 0.00 a	282.89 ± 14.05	292.19 ± 26.88 bc
STR_H_57	428 ± 42 b	235.33 ± 13.54 bc	3.74 ± 0.10 b	0.05 ± 0.01 a	266.79 ± 15.09	270.35 ± 16.99 bc

Data are presented as mean ± SD. Different lowercase letters within the same column indicate significant differences among groups (*p* < 0.05).

**Table 2:** Alpha diversity indices of experimental water microbial communities under mature biofloc and *Vibrio harveyi* treatments.

		Chao1	Sobs	Shannon	Simpson
Sample	NN	511.63 ± 29.74 ab	434.67 ± 25.14 ab	3.5 ± 0.48 ab	0.12 ± 0.08 b
	NV	388.29 ± 30.88 b	310.33 ± 23.4 b	2.08 ± 0.13 b	0.36 ± 0.01 a
	BN	703.38 ± 13.26 a	602.33 ± 29.48 a	3.8 ± 0.39 a	0.11 ± 0.04 b
	BV	463.08 ± 118.18 b	362.33 ± 101.44 b	2.17 ± 0.54 b	0.33 ± 0.07 a
Significance of main effects (Two-way ANOVA)	Biofloc	0.068	0.083	0.658	0.756
	*Vibrio*	0.021	0.011	0.006	0.003
	Biofloc × *Vibrio*	0.382	0.328	0.799	0.941

Data are presented as mean ± SD. Different lowercase letters within the same column indicate significant differences among groups (*p* < 0.05).

## Data Availability

The original contributions presented in this study are included in the article/Supplementary Material. The raw 16S rDNA amplicon sequencing reads generated in this study are available on request from the corresponding author.

## Supplementary Materials

The following supporting information can be downloaded at https://www.mdpi.com/article/10.3390/microorganisms14102349/s1. Table S1: Nitrate nitrogen concentrations at selected cultivation days in biofloc systems; Table S2: Relative abundances (%) of dominant microbial phyla in the STR group under different salinity conditions at various time points; Table S3: Richness and diversity indices of microbial communities in water during biofloc cultivation using brown sugar and shrimp feed as carbon and nitrogen sources; Table S4: Dominant phyla and their relative abundances (%) in SUG_L and SUG_H groups; Table S5: Alpha diversity indices of gut microbial communities under mature biofloc and *Vibrio harveyi* treatments; Figure S1: Comparison of dominant bacterial genera in biofloc systems cultured with rice straw and ammonium chloride as carbon and nitrogen sources (genus level). Statistical significance was assessed using the Kruskal–Wallis H test, with *p* values shown on the right. Asterisks indicate significant differences (*p* < 0.05) among groups for specific genera; Figure S2: Microbial community composition and structure under different salinity conditions using brown sugar and shrimp feed as carbon and nitrogen sources. (A) Principal component analysis (PCA) of microbial communities at the genus level under low-salinity (SUG_L) and high-salinity (SUG_H) conditions. (B) Student’s *t*-test bar plot showing significant differences in genus-level microbial abundances between SUG_L and SUG_H groups, with 95% confidence intervals. (C) Relative abundances of dominant nitrogen-cycling genera (>1%) under different salinity conditions. Asterisks indicate significant differences between groups (* *p* < 0.05, ** *p* < 0.01); Figure S3: Gut microbiota composition of *P. vannamei* under biofloc inoculation and *Vibrio harveyi* challenge (genus level). (A) Principal component analysis (PCA) plot showing genus-level microbial community composition across four treatments. The percentage of variance explained by PC1 and PC2 is indicated on the axes. (B) Heatmap depicting relative abundances of dominant bacterial genera in shrimp gut. Color intensity corresponds to the relative abundance of each genus. (C) Bar plot illustrating the relative abundances (%) of nitrogen-cycling bacteria across treatments. Asterisks (*) indicate statistically significant differences (*p* < 0.05) between groups; Figure S4: Microbial composition of *P. vannamei* hepatopancreas and muscle under biofloc inoculation and *Vibrio harveyi* challenge (genus level). (A) Relative abundances of dominant bacterial genera in shrimp hepatopancreas. Genera with less than 1% relative abundance are grouped under “others”. (B) Comparison of selected genera in hepatopancreas across treatments. Significant differences (*p* < 0.05) are marked. (C) Genus-level relative abundances in shrimp muscle tissue. Low-abundance genera are grouped under “others”. (D) Comparison of key muscle-associated bacterial genera. Error bars indicate mean ± SD (*n* = 3), with significant differences (*p* < 0.05) marked.
